# Validation of Foot and Ankle Ability Measure (FAAM) and the Foot and Ankle Outcome Score (FAOS) in individuals with chronic ankle instability: a cross-sectional observational study

**DOI:** 10.1186/s13018-022-02925-9

**Published:** 2022-01-21

**Authors:** Antonio Manoel Goulart Neto, Nicola Maffulli, Filippo Migliorini, Fábio Sprada de Menezes, Rodrigo Okubo

**Affiliations:** 1 Department of Physiotherapy, State University of Santa Catarina, 358 Pascoal Simone Street, Florianopolis, SC 88080350 Brazil; 2 Department of Orthopaedics, School of Medicine, Surgery and Dentistry, University of Salerno, 132 Via Giovanni Paolo II, 84084 Salerno, Italy; 3 School of Pharmacy and Bioengineering, Faculty of Medicine, Keele University, Newcastle, ST5 5BG UK; 4 Centre for Sports and Exercise Medicine at Queen, Mary University of London, Mile End Rd, Bethnal Green, London, E1 4NS UK; 5 Department of Orthopaedic, Trauma, and Reconstructive Surgery, RWTH University Clinic Aachen, Pauwelsstraße 30, 52074 Aachen, Germany; 6 Physiotherapy Postgraduate Program, State University of Santa Catarina, 358 Pascoal Simone Street, Florianopolis, SC 88080350 Brazil

**Keywords:** Ankle, Joint instability, Postural control, Chronic ankle instability

## Abstract

**Background:**

Ankle sprain is the most common lower limb injury in physically active individuals. Loss of function, decreased postural control (PC), strength deficit, and reduced range of motion (ROM) are common after acute lateral ankle sprains. Some patients experienced long lasting symptoms, with recurrent sprains, and episodes of giving-way: a condition known as chronic ankle instability (CAI). Evaluating the function in patients with CAI in the clinical environment is important to identify the severity of the condition, in addition to allowing to assess the effectiveness of a given treatment. The aim of this study was to investigate the validation of the Foot and Ankle Ability Measure (FAAM) and the Foot and Ankle Outcome Score (FAOS) in terms of muscle strength, PC and ROM in adults with CAI.

**Methods:**

This is a cross-sectional study. Individuals with CAI aged between 18 and 45 years were eligible. Individuals with CAI were identified using the Identification of Functional Ankle Instability (IdFAI). All patients filled in the FAAM and FAOS scores. Muscle strength was assessed by manual dynamometry, ROM by the Lunge test, PC by computerized posturography, modified Star Excursion Balance Test (mSEBT) and modified Balance Error Score System (mBESS).

**Results:**

50 participants were enrolled in the present study. The mean age of the patients was 27.2 ± 6.3 years, and the mean body mass index was 26.4 ± 4.8 kg/m^2^. 58% (29 of 50) were men and 42% (21 of 50) women. 18 individuals had unilateral (36%) and 32 bilateral (64%) CAI. The results of FAAM were associated with MCT, mSEBT, invertor muscles strength, plantar flexor muscles strength, dorsiflexor muscles strength, and external hip rotator muscles strength (*P* < 0.05). The results of FAOS were associated with mSEBT, invertor muscles strength, plantar flexor muscles strength, dorsiflexor muscles strength, evertor muscles strength, and external hip rotator muscles strength, and mBEES (*P* < 0.05).

**Conclusion:**

Both the FAAM and FAOS demonstrated validity to evaluate postural control and muscle strength in patients with CAI, while no association was found in relation to ankle dorsiflexion.

**Supplementary Information:**

The online version contains supplementary material available at 10.1186/s13018-022-02925-9.

## Background

Ankle sprain is the most common lower limb injury in physically active individuals [1]. About 70% of the population experienced one lateral ankle sprain during lifetime [2, 3]. This injury is associated with high costs and long absence from work and recreational activities [1, 4]. Swelling, pain, loss of function, decreased postural control (PC), strength deficit, and reduced range of motion (ROM) are common after an acute lateral ankle sprain [1, 5]. Normally, these symptoms last up to some months [6]. However, some patients experienced long lasting symptoms, with recurrent sprains, and episodes of giving-way: a condition known as chronic ankle instability (CAI) [7–9]. Patients with CAI present alterations of motor patterns, impaired the quality of life (QoL), and may develop early onset osteoarthritis [2, 6, 10–13]. After the development of CAI, 72% of individuals do not return to physical activities at the same previous level, and 6% are unable to return to work [14]. Some studies show decreased function in individuals with CAI compared to those without, in addition to a higher risk of developing posttraumatic osteoarthrosis, lower quality of life and decreased physical activity [2, 4, 15]. Evaluating physical function in patients with CAI in the clinical environment is extremely important to identify the severity of the condition and plat the treatment, in addition to allowing to assess the effectiveness of a given treatment [12].

Several instruments measure the function of the ankle/foot joint complex, but only two are suggested by the International Ankle Consortium to evaluate the functional limitations of individuals with CAI: the Foot and Ankle Ability Measure (FAAM) and the Foot and Ankle Outcome Score (FAOS). The FAAM has 29 items, scored between 0 and 4, divided into two subscales: activities of daily living (21 items) and sports (8 items) [16, 17, 42]. For score analysis, the percentage of each subscale is used separately [17]. FAOS consists of 42 items, with scores between 0 and 4, subdivided into 5 subscales: pain (9 items), other symptoms (7 items), activities of daily living (17 items), sports and recreations (5 items), and quality of life related to ankle and foot (4 items) [17, 18, 45]. Although these measures are subjective and based on the individual's perception of his or her condition, they provide important information regarding ankle-related physical limitation and disability [19].

Currently, the variables associated with the development of CAI are still controversial. Greater knowledge of predisposing factors for CAI is essential to previously identify patients and adopt prompt precautions. The purpose of this study was to investigate the validation of the Foot and Ankle Ability Measure (FAAM) and the Foot and Ankle Outcome Score (FAOS) in terms of muscle strength, postural control (PC), and range of motion (ROM) in adults with CAI.

## Methods

### Study design

The present study was conducted according to the Consolidated Standards of Reporting Trials: the CONSORT statement [20]. This study was approved to the ethics committee and research on human beings at the State University of Santa Catarina (ID 2.799.961), and followed the principles expressed in the Declaration of Helsinki. All patients were free to participate and able to understand the nature of their treatment, providing written consent to use their data for research purposes.

### Participant recruitment

An analytical, cross-sectional study was conducted between January and November 2019 in individuals with CAI at the Department of Physiotherapy of the State University of Santa Catarina, Santa Catarina, Brazil. Individuals were recruited through social networks and posters in places of great circulation in the Florianopolis region, Brazil. The criteria for sample selection followed the recommendations of the International Ankle Consortium [21]. The inclusion criteria were: (1) age between 18 and 45 years, (2) history of ankle sprain, (3) symptoms longer than 12 months, (4) physical activity interruption for at least one day (5) last painful ankle episode within the past 3 months, (6) at last two episode of “giving-way” without resulting in sprain in the last 6 months, (7) instability confirmed by the Identification of Functional Ankle Instability (IdFAI) questionnaire ≥ 11 points [43, 44]. The exclusion criteria were: (1) previous lower limb surgery, (2) previous fractures which required realignment, (3) previous acute musculoskeletal injury to the lower limbs in the last three months.

### Study protocol

**Fig. 1:**
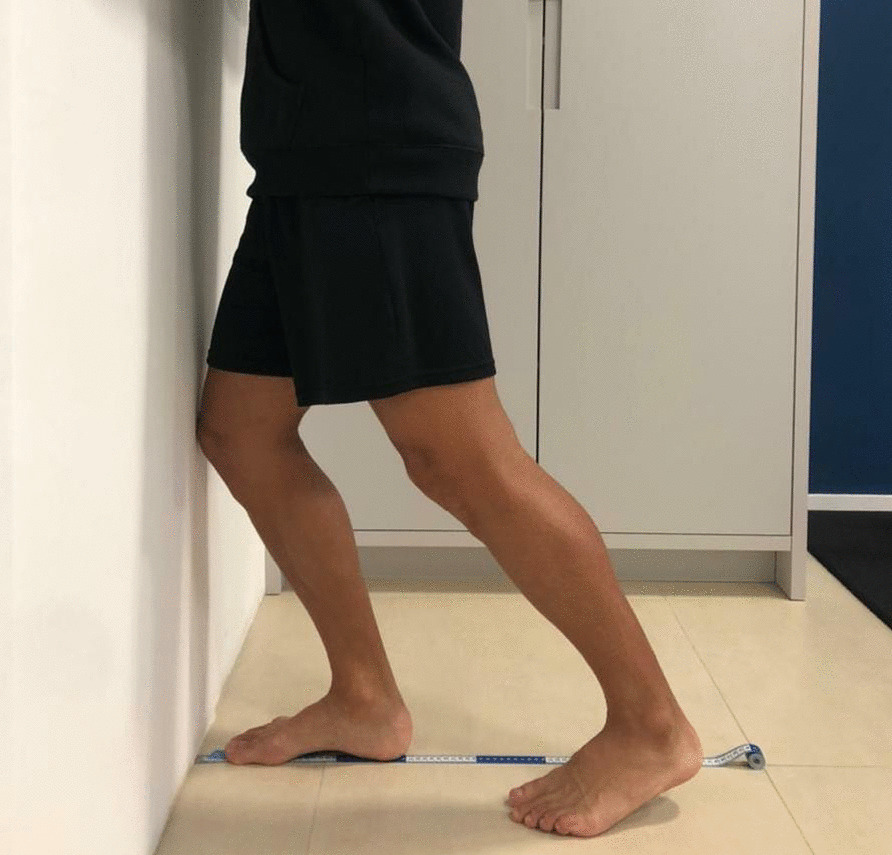
Lunge test

Patient demographic variables were collected: gender, height, age, previous number of sprains, previous treatments, IdFAI questionnaire. Eligible patients were invited to complete the functional evaluations using two questionnaires: the Foot and Ankle Ability Measure (FAAM), which assesses the functional limitation of the foot and ankle [16, 17], and the Foot and Ankle Outcome Score (FAOS), which is based on the patient's perception of the difficulties encountered in relation to the ankle and foot [18]. According to the International Ankle Consortium, values of the activities of day living < 90% and < 80% of the sport subscales of the FAAM score, and < 75% of FAOS score in at least three categories, were considered as not satisfactory. Patients were invited to our institution to assess PC, muscle strength and ankle dorsiflexion. PC was evaluated by: (1) Motor Control Test *(*MCT) using a dynamic computerized posturography (SMART EquiTest, NeuroCom International Inc.); (2) modified Star Excursion Balance Test (mSEBT) to assess the dynamic PC [22]; and (3) modified Balance Error Scoring System (mBESS) to assess the static PC [23]. To assess the muscle strength of invertor, evertor, plantar flexors, dorsiflexor and external hip rotators, a Lafayette® manual dynamometer (Lafayette Instrument, model 01,165, Lafayette, US) was used. Participants were instructed how to practice the tests with 50%, 75%, and 100% of the muscle strength against resistance, and how to produce three valid attempts of isometric contraction for 5 s, with a 15-s rest period between attempts [24]. Results were expressed as the mean of the three attempts for each muscle group in terms of Kilogram force (Kgf). The Lunge test was used to assess ankle dorsiflexion (Fig. 1) [25]. Patients were invited to repeat the test three times. The measure used was the distance between the hallux and the wall in cm of the longest range achieved during the attempts.

**Fig. 2:**
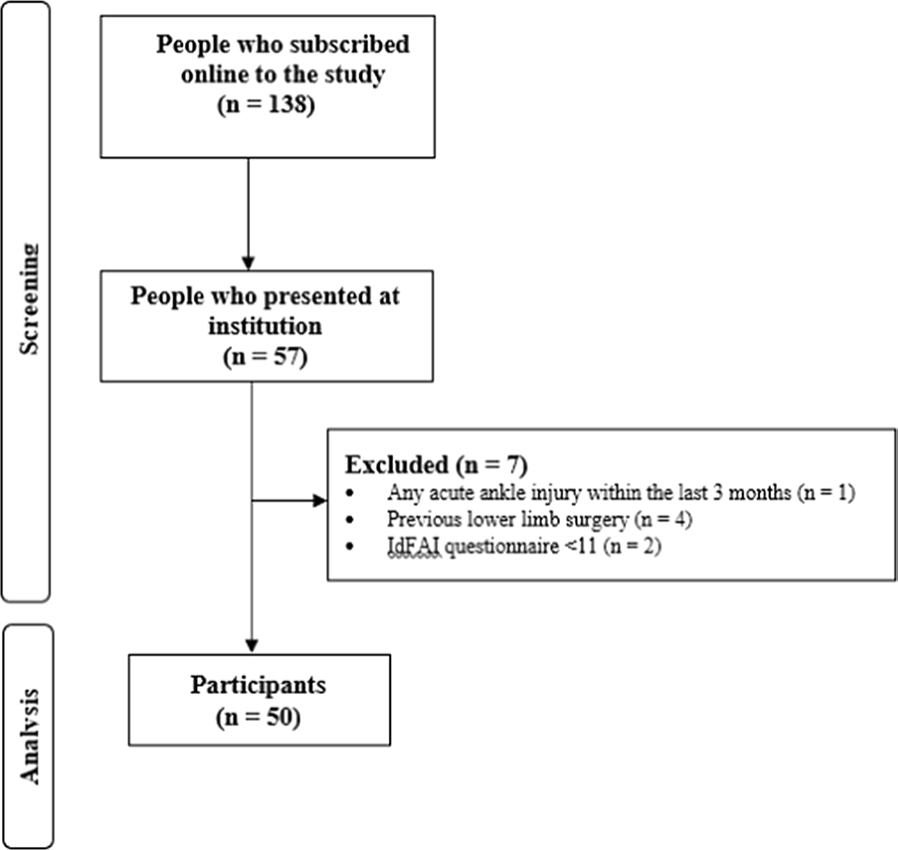
Diagram of the enrolment process

### Data analysis

The statistical analyses were performed using the software STATA /MP (StataCorp, College Station, Texas). A multiple linear model regression analysis through the Pearson Product-Moment Correlation Coefficient (*r*) was used. The Cauchy–Schwarz formula was used for inequality: + 1 is considered as positive linear correlation, while and − 1 a negative one. Values of 0.1 <| *r* |< 0.3, 0.3 <| *r* |< 0.5, and | *r* |> 0.5 were considered to have small, medium, and strong correlation, respectively. The overall significance was performed through the *χ*^2^ test, with values of *P* < 0.05 considered statistically significant.

## Results

### Patient recruitment

A total of 138 people registered themselves to the online website recruitment. Of them, 57 presented to our institution. Seven people were excluded: no acute ankle injury within the last 3 months (*n* = 1), previous lower limb surgery (*n* = 4), IdFAI questionnaire < 11 (*n* = 2). This left 50 participants in the present study (Fig. 2).

The mean age of the sample was 27.2 ± 6.3 years, and the mean body mass index (BMI) was 26.4 ± 4.8 kg/m^2^. 58% (29 of 50) were men and 42% (21 of 50) women. 18 individuals had unilateral (36%) and 32 bilateral (64%) CAI. Demographic characteristics are shown in greater detail in Table 1.

**Table 1:** Participant demographics

Participants (*N* = 50)	
Age (years)	27.2 ± 6.3
Weight (kg)	79.0 ± 16.4
Height (cm)	172.4 ± 9.2
BMI	26.4 ± 4.8
IdFAI	27.2 ± 7.4
FAAM ADL	82.4 ± 15.4
FAAM sport	69.8 ± 19.6
FAOS symptom	69.2 ± 17.8
FAOS pain	81.1 ± 14.5
FAOS ADL	88.4 ± 14.0
FAOS sport	70.7 ± 24.9
FAOS quality of life	52.4 ± 24.2

BMI, body mass index; IdFAI, Identification of Functional Ankle Instability; FAAM, Foot and Ankle Ability Measure; ADL, activities of daily life; FAOS, Foot and Ankle Outcome Score

### Outcomes of interest

FAAM—activities of daily living (ADL) was associated with MCT (*P* = 0.01), mSEBT (*P* = 0.004), invertor muscles strength (*P* = 0.003), plantar flexor muscles strength (*P* = 0.007), dorsiflexor muscles strength (*P* = 0.04), and external hip rotator muscles strength (*P* = 0.04). FAAM—Sports was associated with invertor muscles strength (*P* = 0.002), plantar flexor muscles strength (*P* = 0.03). FAOS—Symptoms was associated with mSEBT (*P* = 0.03), invertor muscles strength (*P* = 0.006), plantar flexor muscles strength (*P* = 0.01), dorsiflexor muscles strength (*P* = 0.01). FAOS—Pain was associated with mSEBT (*P* = 0.002), invertor muscles strength (*P* = 0.0002), evertor muscles strength (*P* = 0.04), plantar flexor muscles strength (*P* = 0.001), dorsiflexor muscles strength (*P* = 0.003), and external hip rotator muscles strength (*P* = 0.03). FAOS-ADL was associated with MCT (*P* = 0.002), mSEBT (*P* = 0.0001), mBEES (*P* = 0.04), invertor muscles strength (*P* = 0.001), plantar flexor muscles strength (*P* = 0.005), dorsiflexor muscles strength (*P* = 0.02), and external hip rotator muscles strength (*P* = 0.03). FAOS—Sports and recreation was associated with MCT (*P* = 0.02), mSEBT (*P* = 0.01), invertor muscles strength (*P* = 0.0006), plantar flexor muscles strength (*P* = 0.006), and dorsiflexor muscles strength (*P* = 0.04). FAOS—QoL was associated with mSEBT (*P* = 0.0001), invertor muscles strength (*P* = 0.0002), evertor muscles strength (*P* = 0.02), plantar flexor muscles strength (*P* = 0.0003), and dorsiflexor muscles strength (*P* = 0.005). The results of the multivariate analyses are shown in greater detail in Table 2.

**Table 2:** Results of the multivariate analysis

Variables	FAAM		FAOS											
	ADL		Sports		Symptoms		Pain		ADL		Sports and recreation		QoL	
Postural control														
MCT	− 0.35	0.01	− 0.18	0.2	− 0.22	0.1	− 0.26	0.06	− 0.43	0.002	− 0.34	0.02	− 0.28	0.05
mSEBT	0.40	0.004	0.28	0.05	0.31	0.03	0.43	0.002	0.51	0.0001	0.34	0.01	0.51	0.0001
mBEES	− 0.26	0.1	− 0.19	0.2	− 0.02	0.9	− 0.27	0.06	− 0.30	0.04	− 0.18	0.2	− 0.26	0.06
Muscle strength														
Invertor	0.42	0.003	0.43	0.002	0.38	0.006	0.50	0.0002	0.44	0.001	0.47	0.0006	0.50	0.0002
Evertor	0.24	0.1	0.24	0.1	0.20	0.2	0.30	0.04	0.26	0.07	0.24	0.09	0.33	0.02
Plantar flexors	0.38	0.007	0.31	0.03	0.36	0.01	0.45	0.001	0.39	0.005	0.38	0.006	0.49	0.0003
Dorsiflexor	0.29	0.04	0.23	0.1	0.29	0.04	0.41	0.003	0.33	0.02	0.29	0.04	0.39	0.005
External hip rotators	0.30	0.04	0.16	0.3	0.21	0.2	0.31	0.03	0.30	0.03	0.26	0.06	0.23	0.1
Ankle dorsiflexion														
Lunge test	0.19	0.2	0.11	0.4	0.13	0.4	0.21	0.1	0.20	0.2	0.19	0.2	0.22	0.1

FAAM, Foot and Ankle Ability Measure; ADL, activities of daily life; QoL, quality of life; FAOS, Foot and Ankle Outcome Score; MCT, Motor Control Test; mSEBT, modified Star Excursion Balance Test; mBESS, modified Balance Error Scoring System

## Discussion

According to the main findings of the present study, both the FAAM and FAOS demonstrated reliability to evaluate postural control and muscle strength in patients with CAI, while no association was found in relation to the dorsiflexion mobility test. The literature presents previous FAAM and FAOS validations, but only validations related to reliability with others scales and/or associated with cross-cultural translations [26–32]. In one of these studies, Matheny et al. [26] showed that normative values of foot and ankle outcome measures did not reflect 100% function. Therefore, we decided to evaluate posturography, strength and mobility outcomes to validate these functional scales, verifying whether some of them are associated with the scores of CAI patients.

Frequently, studies on CAI use the assessments of physical components to try to elucidate how their development and associated factors occur, and/or improve the therapeutic approach, and the use of tools that can measure the function of this population, as an outcome has been growing [2]. Muscle strength, PC and ROM deficits present in CAI contribute to worsening of function in these individuals [2, 25]. In addition, several studies have shown that it is possible to positively modify function in individuals with CAI through interventions focused on improving these deficits, especially with balance training [2]. In this study, we found that FAAM and FAOS are reliabile measure dynamic postural balance, demonstrating moderate to strong association in most of FAAM and FAOS domains. It is worth mentioning the strong association with the ADL and QoL subscales in FAOS, which may be directly linked to the presence of sensory alterations, such as PC deficit [12].

In the literature, strength deficits are consistently present in individuals with CAI [7, 33], and many interventions are based on the improvement of this and other dysfunctions aimed at improving the function of these individuals [34], although there is no evidence to support the use of muscle strengthening as an isolated strategy to improve function in this population [2]. Our results demonstrate reliability of FAAM and FAOS to evaluate functional muscle strength, since they demonstrate a moderate association of muscle strength of invertor and plantiflexors with all subscales of the questionaries and to a lesser degree to the ankle dorsiflexor, external hip rotators and ankle evertor. Even though there is an association between strength and function measures, some studies hypothesise that muscle strength does not play an important role in decreasing function in CAI [35–37], and that more important would be proper activation during functional tasks [2, 35–37]. On the other hand, other studies put forward the idea that the functional loss associated with CAI may result from the limitations imposed by the various deficits present in this population, including the loss of muscle strength [12, 21].

Among the various factors investigated in the literature, ROM is listed as possibly associated with the onset and maintenance of prolonged symptoms [2]. Although some studies demonstrate an association between ROM and function [25, 38] our findings corroborate another study [33] that shows no association between ROM and function (FAOS and FAAM).

CAI is a complex and multifactorial condition, and the perception of function through questionnaires can be influenced not only by biological factors, but also by the social and psychological demands of each individual [7]. The path to the development of deficits in CAI is not well understood, but it is present and may be the main reason for making this population less physically active [11, 39]. Decrease in the level of physical activity may be one of the factors that contribute to maintaining or worsening the deficits over the years [40]. In addition, Lee et al. [41] reported that the unaffected contralateral ankles of individuals with CAI also showed significant decreases in postural stability and neuromuscular control, and one should consider the non-injured limb and the activity of these patients to prevent future events of contralateral sprain. Within the multifactorial and not yet fully understood panorama of this condition, our study reinforces the importance of using specific questionnaires for evaluation and follow-up of patients with CAI, demonstrating that functional worsening is associated with a worsening of muscle strength and PC.

This study has several limitations. Only individuals with CAI aged 18–45 years were included, which potentially increases the risk of selection bias of the present work. The level of physical activity and kinesiophobia, or questionaries related to quality of life were not additionally administered. The outcome variables of interest at the time of evaluation were not adjusted according to the duration of symptoms. Moreover, participants were recruited through social networks and posters in places of great circulation in the Florianopolis region. This methodology of recruitment process may have led to increase the risk of selection bias. Moreover, no control group was included, which may enhance the risk of detection bias.

Despite several studies over many years, the risk factors in CAI are not fully understood. We were unable to identify additional studies which validated functional questionnaires in individuals with CAI. Current practice can benefit from the FAAM and FAOS, as they demonstrated to be valid and available tools to assess postural control and muscle strength in patients with CAI. These questionnaires can be easily used in current clinical practice, are easy to apply and free from specialised instrumentation and equipment.

## Conclusion

Both the FAAM and FAOS demonstrated validity to evaluate the postural control and muscle strength in patients with CAI, while no association was found in relation to ankle dorsiflexion. This study demonstrated that function assessment is an important measure in patients with CAI in the clinical setting. The latter may be useful to identify the severity of instability. Further studies should validate the FAAM and FAOS as potential tools to monitor the efficacy of rehabilitation and postoperative care (Additional files 1 [43], 2 [44], 3 [45], 4 [18], 5 [42], 6 [16]).

## Supplementary Information

**Additional file 1**. Identification of Functional Ankle Instability (IdFAI) questionnaire- English version

**Additional file 2**. Identification of Functional Ankle Instability (IdFAI) questionnaire - Brazilian version.

**Additional file 3**. Foot and Ankle Outcome Score (FAOS) questionnaire - English version.

**Additional file 4**. Foot and Ankle Outcome Score (FAOS) questionnaire - Brazilian version.

**Additional file 5**. Foot and Ankle Ability Measure (FAAM) questionnaire - English version.

**Additional file 6**. Foot and Ankle Ability Measure (FAAM) questionnaire - Brazilian version.

## Data Availability

The datasets used and analysed during the current study are available from the corresponding author on reasonable request.

## Abbreviations

PC: Postural control
ROM: Range of motion
CAI: Chronic ankle instability
QoL: Quality of life
FAAM: Foot and Ankle Ability Measure
FAOS: Foot and Ankle Outcome Score
CONSORT: Consolidated Standards of Reporting Trials
IdFAI: Identification of Functional Ankle Instability
MCT: Motor Control Test
mSEBT: Modified Star Excursion Balance Test
mBESS: Modified Balance Error Scoring System
Kgf: Kilogram force
*r*: Pearson Product-Moment Correlation Coefficient
BMI: Body mass index
ADL: Activities of daily life
