# Supplementary material for: Validation of Foot and Ankle Ability Measure (FAAM) and the Foot and Ankle Outcome Score (FAOS) in individuals with chronic ankle instability: a cross-sectional observational study
Source: J Orthop Surg Res. 2022 Jan 21;17:38. doi: 10.1186/s13018-022-02925-9 (PMC8781557; doi:10.1186/s13018-022-02925-9)
Supplement: Supplementary file 1 — Additional file 1. Identification of Functional Ankle Instability (IdFAI) questionnaire- English version [file 13018_2022_2925_MOESM1_ESM.pdf]

IDENTIFICATION OF FUNCTIONAL ANKLE INSTABILITY (IdFAI)

instructions: this form will be used to categorize your ankle stability status. A separete form should be used for the right and left ankles. Please fill out the form completely and if you have any questions, please ask the administrator. Thank you for your participation.

Please carefully read the following statement: "giving way" is described as a temporary uncontrollable sensation of instability or rolling over of one's ankle

I am completing this form for my RIGHT/LEFT ankle (circle one)

1) Approximately how many times have you sprained your ankle?

____

2) When was the last time you sprained your ankle ?

- Never
- >2 years
- 1 - 2 years
- 6 - 12 months
- 1 - 6 months
- < 1 month

3) If you have seen an athletic trainer, physician, or healthcare provider how did he/she categorize your most serious ankle sprain?

- have not seen someone
- Mild (Grade I)
- Moderate (Grade II)
- Severe (Grade III)

4) If you have ever used crutches or other device, due to an ankle sprain how long did you use it?

- Never used a device
- 1-3 days
- 4-7 days
- 1-2 weeks
- 2-3 weeks
- >3 weeks

5) When was the last time you had "giving way" your ankles?

- Never
- >2 years
- 1-2 years
- 6-12 months
- 1-6 months
- <1 month

6) How often does the "giving way" sensation occur in your ankle?

- Never
- Once a year
- Once a month
- Once a week
- Once a day

7) Tipically when you start to roll over (or "twist") on your ankle can you stop it?

- Never rolled over
- Immediately
- Sometimes
- Unable to stop it

8) Following a typical incident of your ankle rolling over, how soon does it return to normal?

- Never rolled over
- Immediately
- <1 day
- 1-2 days
- >2 days

9) During "activities of daily life" how often does your ankle feel UNSTABLE?

- Never
- Once a year
- Once a month
- Once a week
- Once a day

10) During "Sport/or recreational activities" how often does your ankle feel UNSTABLE?

- Never
- Once a year
- Once a month
- Once a week
- Once a day
