# Supplementary material for: Validation of Foot and Ankle Ability Measure (FAAM) and the Foot and Ankle Outcome Score (FAOS) in individuals with chronic ankle instability: a cross-sectional observational study
Source: J Orthop Surg Res. 2022 Jan 21;17:38. doi: 10.1186/s13018-022-02925-9 (PMC8781557; doi:10.1186/s13018-022-02925-9)
Supplement: Supplementary file 2 — Additional file 2. Identification of Functional Ankle Instability (IdFAI) questionnaire - Brazilian version. [file 13018_2022_2925_MOESM2_ESM.pdf]

IDENTIFICAÇÃO DA INSTABILIDADE FUNCIONAL DE TORNOZELO (IdFAI) - BRAZILIAN VERSION

Instruções: Este formulário será utilizado para classificar o grau de estabilidade do seu tornozelo. Deve ser utilizado um formulário para cada tornozelo, direito e esquerdo. Por favor, preencha o formulário completamente, e se tiver alguma dúvida pergunte ao avaliador. Obrigado pela participação

---

Por favor, leia com atenção a seguinte afirmação: "Falseio" é uma sensação de instabilidade momentânea, podendo ou não, levar a torção de um dos tornozelos.

Estou preenchendo este formulário para meu tornozelo DIREITO/ESQUERDO (circule um)

1) Até hoje, quantas vezes você já torceu o tornozelo?

____

2) Quando foi a última vez que você torceu o tornozelo?

- ◯ Nunca
- ◯ >2 anos
- ◯ 1 - 2 anos
- ◯ 6 - 12 meses
- ◯ 1 - 6 meses
- ◯ < 1 mês

3) Se você já procurou alguma vez um educador físico, médico ou profissional da saúde, como ele/ela classificou a sua entorse de tornozelo mais grave?

- NÃO procurou ninguém
- Leve (Grau I)
- Moderado (Grau II)
- Grave (Grau III)

4)Se você já usou muletas, ou outro dispositivo, devido a uma entorse de tornozelo, por quanto tempo você utilizou?

- Nunca usou
- 1-3 dias
- 4-7 dias
- 1-2 semanas
- 2-3 semanas
- >3 semanas

5) Quando foi a última vez que você sentiu "falseio" no seu tornozelo?

- Nunca
- >2 anos
- 1-2 anos
- 6-12 meses
- 1-6 meses
- <1 mes

6)Com que frequência a sensação de "falseio"ocorre no seu tornozelo?

- Nunca
- Umas vez por ano
- Uma vez por mês
- Uma vez por semana
- Uma vez por dia

7)Tipicamente, quando sente que seu tornozelo irá torcer, você consegue evitar?

- Nunca torceu
- imediatamente
- Às vezes
- Incapaz de evitar

8) Após um incidente típico de torção do seu tornozelo, quanto tempo depois ele leva para voltar ao "normal"?

- Nunca torceu
- Imediatamente
- <1 dia
- 1-2 dias
- >2 dias

9) Durante "atividades de vida diária" com que frequência você sente seu tornozelo INSTÁVEL?

- Nunca
- Uma vez por ano
- Uma vez por mês
- Uma vez por semana
- Uma vez por dia

10) Durante "atividades esportivas/ou recreativas" com que frequência você sente seu tornozelo INSTÁVEL?

- Nunca
- Uma vez por ano
- Uma vez por mês
- Uma vez por semana
- Uma vez por dia
