# Supplementary material for: Validation of Foot and Ankle Ability Measure (FAAM) and the Foot and Ankle Outcome Score (FAOS) in individuals with chronic ankle instability: a cross-sectional observational study
Source: J Orthop Surg Res. 2022 Jan 21;17:38. doi: 10.1186/s13018-022-02925-9 (PMC8781557; doi:10.1186/s13018-022-02925-9)
Supplement: Supplementary file 3 — Additional file 3. Foot and Ankle Outcome Score (FAOS) questionnaire - English version. [file 13018_2022_2925_MOESM3_ESM.pdf]

FOOT AND ANKLE OUTCOME SCORE (FAOS)

INSTRUCTIONS: This survey asks for your view about your foot/ankle. This information will help us keep track of how you feel about your foot/ankle and how well you are able to do your usual activities. Answer every question by ticking the appropriate box, only one box for each question. If you are unsure about how to answer a question, please give the best answer you can.

SYMPTOMS

These questions should be answered thinking of your foot/ankle symptoms during the last week.

S1 - Do you have swelling in your foot/ankle?

- Never
- Rarely
- Sometimes
- Often
- Always

S2 - Do you feel grinding, hear clicking or any other type of noise when your foot/anklemoves?

- Never
- Rarely
- Sometimes
- Often
- Always

S3 - Does your foot/ankle catch or hang up when moving?

- Never
- Rarely
- Sometimes
- Often
- Always

S4 - Can you straighten your foot/ankle fully?

- Always
- Often
- Sometimes
- Rarely
- Never

S5 - Can you bend your foot/ankle fully?

- Always
- Often
- Sometimes
- Rarely
- Never

STIFFNESS

The following questions concern the amount of joint stiffness you have experienced during the last week in your foot/ankle. Stiffness is a sensation of restriction or slowness in the ease with which you move your joints.

S6 - How severe is your foot/ankle stiffness after first wakening in the morning?

- None
- Mild
- Moderate
- Severe
- Extreme

S7 - How severe is your foot/ankle stiffness after sitting, lying or resting later in theday?

- None
- Mild
- Moderate
- Severe
- Extreme

PAIN

P1 - How often do you experience foot/ankle pain?

- Never
- Monthly
- Weekly
- Daily
- Always

What amount of foot/ankle pain have you experienced the last week during thefollowing activities?

P2 - Twisting/pivoting on your foot/ankle

[illegible]

- None
- Mild
- Moderate
- Severe
- Extreme

P3 - Straightening foot/ankle fully

- None
- Mild
- Moderate
- Severe
- Extreme

P4 - Bending foot/ankle fully

- None
- Mild
- Moderate
- Severe
- Extreme

P5 - Walking on flat surface

- None
- Mild
- Moderate
- Severe
- Extreme

P6 - Going up or down stairs

- None
- Mild
- Moderate
- Severe
- Extreme

P7 - At night while in bed

- None
- Mild
- Moderate
- Severe
- Extreme

P8 - Sitting or lying

- None
- Mild
- Moderate
- Severe
- Extreme

P9 - Standing upright

- None
- Mild
- Moderate
- Severe
- Extreme

FUNCTION, DAILY LIVING

The following questions concern your physical function. By this we mean your ability to move around and to look after yourself. For each of the following activities please indicate the degree of difficulty you have experienced in the last week due to your foot/ankle.

A1 - Descending stairs

- None
- Mild
- Moderate
- Severe
- Extreme

A2 - Ascending stairs

- None
- Mild
- Moderate
- Severe
- Extreme

A3 - Rising from sitting

- None
- Mild
- Moderate
- Severe
- Extreme

A4 - Standing

- None
- Mild
- Moderate
- Severe
- Extreme

A5 - Bending to floor/pick up an object

- None
- Mild
- Moderate
- Severe
- Extreme

A6 - Walking on flat surface

- None
- Mild
- Moderate
- Severe
- Extreme

A7 - Getting in/out of car

- None
- Mild
- Moderate
- Severe
- Extreme

A8 - Going shopping

- None
- Mild
- Moderate
- Severe
- Extreme

A9 - Putting on socks/stockings

- None
- Mild
- Moderate
- Severe
- Extreme

A10 - Rising from bed

- None
- Mild
- Moderate
- Severe
- Extreme

A11 - Taking off socks/stockings

- None
- Mild
- Moderate
- Severe
- Extreme

A12 - Lying in bed (turning over, maintaining foot/ankle position)

- None
- Mild
- Moderate
- Severe
- Extreme

A13 - Getting in/out of bath

- None
- Mild
- Moderate
- Severe
- Extreme

A14 - Sitting

- None
- Mild
- Moderate
- Severe
- Extreme

A15 - Getting on/off toilet

- None
- Mild
- Moderate
- Severe
- Extreme

A16 - Heavy domestic duties (moving heavy boxes, scrubbing floors, etc)

- None
- Mild
- Moderate
- Severe
- Extreme

A17 - Light domestic duties (cooking, dusting, etc)

- None
- Mild
- Moderate
- Severe
- Extreme

FUNCTION, SPORTS AND RECREATIONAL ACTIVITIES

The following questions concern your physical function when being active on a higher level. The questions should be answered thinking of what degree of difficulty you have experienced during the last week due to your foot/ankle.

SP1 - Squatting

- None
- Mild
- Moderate
- Severe
- Extreme

SP2 - Running

- None
- Mild
- Moderate
- Severe
- Extreme

SP3 - Jumping

- None
- Mild
- Moderate
- Severe
- Extreme

SP4 - Twisting/pivoting on your injured foot/ankle

- None
- Mild
- Moderate
- Severe
- Extreme

SP5 - Kneeling

- None
- Mild
- Moderate
- Severe
- Extreme

QUALITY OF LIFE

Q1 - How often are you aware of your foot/ankle problem?

- Never
- Monthly
- Weekly
- Daily
- Constantly

Q2 - Have you modified your life style to avoid potentially damaging activitiesto your foot/ankle?

- Not at all
- Mildly
- Moderatly
- Severely
- Totally

Q3 - How much are you troubled with lack of confidence in your foot/ankle?

- Not at all
- Mildly
- Moderatly
- Severely
- Extremely

Q4 - In general, how much difficulty do you have with your foot/ankle?

- None
- Mild
- Moderate
- Severe
- Extreme
