# Supplementary material for: Validation of Foot and Ankle Ability Measure (FAAM) and the Foot and Ankle Outcome Score (FAOS) in individuals with chronic ankle instability: a cross-sectional observational study
Source: J Orthop Surg Res. 2022 Jan 21;17:38. doi: 10.1186/s13018-022-02925-9 (PMC8781557; doi:10.1186/s13018-022-02925-9)
Supplement: Supplementary file 4 — Additional file 4. Foot and Ankle Outcome Score (FAOS) questionnaire - Brazilian version. [file 13018_2022_2925_MOESM4_ESM.pdf]

FOOT AND ANKLE OUTCOME SCORE (FAOS) - BRAZILIAN VERSION

Para avaliação da função e sintomas do tornozelo e pé

SINTOMAS

S1 - Qual o grau de rigidez do seu pé/tornozelo logo quando você acorda?

- Nenhuma
- Leve
- Moderada
- Acentuada
- Extrema

S2 - Qual o grau de rigidez após sentar, deitar ou ao descansar mais tarde durante o dia?

- Nenhuma
- Leve
- Moderada
- Acentuada
- Extrema

S3 - Você tem inchaço no seu pé/tornozelo?

- Nunca
- Raramente
- Às vezes
- Frequentemente
- Sempre

S4 - Você sente ranger, estalar ou qualquer outro tipo de som quando movimenta o pé?

- Nunca
- Raramente
- Às vezes
- Frequentemente
- Sempre

S5 - O seu pé trava ou fica bloqueado aos movimentos?

- Nunca
- Raramente
- Às vezes
- Frequentemente
- Sempre

S6 - Você consegue forçar o seu pé completamente para baixo?

- Sempre
- Frequentemente
- Às vezes
- Raramente
- Nunca

S7 - Você consegue forçar o seu pé completamente para cima?

- Sempre
- Frequentemente
- Às vezes
- Raramente
- Nunca

DOR

P1 - Qual a frequência que você sente dor no pé ou tornozelo?

- Nunca
- Mensalmente
- Semanalmente
- Diariamente
- Sempre

Qual a intensidade de dor que você sentiu na última semana durante as seguintes atividades?

P2 - Rodando sobre o seu pé ou tornozelo

- Nenhuma
- Leve
- Moderada
- Acentuada
- Extrema

P3 - Forçando o pé completamente para baixo

- Nenhuma
- Leve
- Moderada
- Acentuada
- Extrema

P4 - Forçando o pé completamente para cima

- Nenhuma
- Leve
- Moderada
- Acentuada
- Extrema

P5 - Andando em superfície plana

- Nenhuma
- Leve
- Moderada
- Acentuada
- Extrema

P6 - Subindo ou descendo escadas

- Nenhuma
- Leve
- Moderada
- Acentuada
- Extrema

P7 - Em repouso na cama

- Nenhuma
- Leve
- Moderada
- Acentuada
- Extrema

P8 - Ao sentar-se/deitar-se

- Nenhuma
- Leve
- Moderada
- Acentuada
- Extrema

P9 - Em pé

- Nenhuma
- Leve
- Moderada
- Acentuada
- Extrema

ATIVIDADES DE VIDA DIÁRIA

Qual a dificuldade que você sentiu na ultima semana:

A1 - Descendo escadas

- Nenhuma
- Leve
- Moderada
- Acentuada
- Extrema

A2 - Subindo escadas

- Nenhuma
- Leve
- Moderada
- Acentuada
- Extrema

A3 - Levantando-se a partir da posição sentada

- Nenhuma
- Leve
- Moderada
- Acentuada
- Extrema

A4 - Em pé

- Nenhuma
- Leve
- Moderada
- Acentuada
- Extrema

A5 - Curvando-se para pegar um objeto no chão

- Nenhuma
- Leve
- Moderada
- Acentuada
- Extrema

A6 - Andando em superfícies planas

- Nenhuma
- Leve
- Moderada
- Acentuada
- Extrema

A7 - Entrando e saindo do carro

- Nenhuma
- Leve
- Moderada
- Acentuada
- Extrema

A8 - Indo às compras

- Nenhuma
- Leve
- Moderada
- Acentuada
- Extrema

A9 - Colocando meias

- Nenhuma
- Leve
- Moderada
- Acentuada
- Extrema

A10 - Levantando-se da cama

- Nenhuma
- Leve
- Moderada
- Acentuada
- Extrema

A11 - Tirando as meias

- Nenhuma
- Leve
- Moderada
- Acentuada
- Extrema

A12 - Virando-se na cama, mantendo a mesma posição do tornozelo/pé

- Nenhuma
- Leve
- Moderada
- Acentuada
- Extrema

A13 - Entrando e saindo do banho

- Nenhuma
- Leve
- Moderada
- Acentuada
- Extrema

A14 - Sentando

- Nenhuma
- Leve
- Moderada
- Acentuada
- Extrema

A15 - Sentando e levantando do vaso sanitário

- Nenhuma
- Leve
- Moderada
- Acentuada
- Extrema

A16 - Realizando tarefas domésticas pesadas (deslocando caixas pesadas, esfregando o chão, etc)

- Nenhuma
- Leve
- Moderada
- Acentuada
- Extrema

A17 - Realizando tarefas domésticas leves (cozinhando, varrendo, etc)

- Nenhuma
- Leve
- Moderada
- Acentuada
- Extrema

ESPORTES E RECREAÇÕES FUNCIONAIS

Qual a dificuldade que você sentiu nesta ultima semana:

SP1 - Agachando

- Nenhuma
- Leve
- Moderada
- Acentuada
- Extrema

SP2 - Correndo

- Nenhuma
- Leve
- Moderada
- Acentuada
- Extrema

SP3 - Pulando

- Nenhuma
- Leve
- Moderada
- Acentuada
- Extrema

SP4 - Mudando de direção sobre o seu tornozelo/pé lesionado

- Nenhuma
- Leve
- Moderada
- Acentuada
- Extrema

SP5 - Ajoelhando-se

- Nenhuma
- Leve
- Moderada
- Acentuada
- Extrema

QUALIDADE DE VIDA EM RELAÇÃO AO PÉ E TORNOZELO

Q1 - Com que frequência que você tem percebido os problemas do seu tornozelo/pé?

- Nunca
- Mensalemnte
- Semanalmente
- Diariamente
- Sempre

Q2 - Você tem modificado seu estilo de vida para evitar atividades potencialmente danosas para o seu pé e tornozelo?

- Não
- Um pouco
- Moderadamente
- Muito
- Totalmente

Q3 - O quanto você está incomodado com a falta de confiança no seu tornozelo/pé?

- Não
- Um pouco
- Moderadamente
- Muito
- Totalmente

Q4 - No geral, quanto de dificuldade você tem com o seu pé/tornozelo?

- Nenhuma
- Leve
- Moderada
- Acentuada
- Extrema
