# Supplementary material for: Validation of Foot and Ankle Ability Measure (FAAM) and the Foot and Ankle Outcome Score (FAOS) in individuals with chronic ankle instability: a cross-sectional observational study
Source: J Orthop Surg Res. 2022 Jan 21;17:38. doi: 10.1186/s13018-022-02925-9 (PMC8781557; doi:10.1186/s13018-022-02925-9)
Supplement: Supplementary file 5 — Additional file 5. Foot and Ankle Ability Measure (FAAM) questionnaire - English version. [file 13018_2022_2925_MOESM5_ESM.pdf]

FOOT AND ANKLE ABILITY MEASURE (FAAM)- Activities of Daily Living Subscale

Please Answer every question with one response that most closely describes your condition within the past week.
If the activity in question is limited by something other than your foot or ankle mark "Not Applicable" (N/A).

Because of your foot and ankle how much difficulty do you have with:

1. Standing

- No Difficulty
- Slight Difficulty
- Moderate Difficulty
- Extreme Difficulty
- Unable to do
- N/A

2. Walking on even Ground

- No Difficulty
- Slight Difficulty
- Moderate Difficulty
- Extreme Difficulty
- Unable to do
- N/A

3. Walking on even ground without shoes

- No Difficulty
- Slight Difficulty
- Moderate Difficulty
- Extreme Difficulty
- Unable to do
- N/A

4. Walking up hills

- No Difficulty
- Slight Difficulty
- Moderate Difficulty
- Extreme Difficulty
- Unable to do
- N/A

5. Walking down hills

- No Difficulty
- Slight Difficulty
- Moderate Difficulty
- Extreme Difficulty
- Unable to do
- N/A

6. Going up stairs

- No Difficulty
- Slight Difficulty
- Moderate Difficulty
- Extreme Difficulty
- Unable to do
- N/A

7. Going down stairs

- No Difficulty
- Slight Difficulty
- Moderate Difficulty
- Extreme Difficulty
- Unable to do
- N/A

8. Walking on uneven ground

- No Difficulty
- Slight Difficulty
- Moderate Difficulty
- Extreme Difficulty
- Unable to do
- N/A

9. Stepping up and down curbs

- No Difficulty
- Slight Difficulty
- Moderate Difficulty
- Extreme Difficulty
- Unable to do
- N/A

10. Squatting

- No Difficulty
- Slight Difficulty
- Moderate Difficulty
- Extreme Difficulty
- Unable to do
- N/A

11. Coming up on your toes

- No Difficulty
- Slight Difficulty
- Moderate Difficulty
- Extreme Difficulty
- Unable to do
- N/A

12. Walking initially

- No Difficulty
- Slight Difficulty
- Moderate Difficulty
- Extreme Difficulty
- Unable to do
- N/A

13. Walking 5 minutes or less

- No Difficulty
- Slight Difficulty
- Moderate Difficulty
- Extreme Difficulty
- Unable to do
- N/A

14. Walking approximately 10 minutes

- No Difficulty
- Slight Difficulty
- Moderate Difficulty
- Extreme Difficulty
- Unable to do
- N/A

15. Walking 15 minutes or greater

- No Difficulty
- Slight Difficulty
- Moderate Difficulty
- Extreme Difficulty
- Unable to do
- N/A

16. Home responsibilities

- No Difficulty
- Slight Difficulty
- Moderate Difficulty
- Extreme Difficulty
- Unable to do
- N/A

17. Activities of daily living

- No Difficulty
- Slight Difficulty
- Moderate Difficulty
- Extreme Difficulty
- Unable to do
- N/A

18. Personal care

- No Difficulty
- Slight Difficulty
- Moderate Difficulty
- Extreme Difficulty
- Unable to do
- N/A

19. Light to moderate work (standing, walking)

- No Difficulty
- Slight Difficulty
- Moderate Difficulty
- Extreme Difficulty
- Unable to do
- N/A

20. Heavy work (push/pulling,climbing, carrying)

- No Difficulty
- Slight Difficulty
- Moderate Difficulty
- Extreme Difficulty
- Unable to do
- N/A

21. Recreational activities

- No Difficulty
- Slight Difficulty
- Moderate Difficulty
- Extreme Difficulty
- Unable to do
- N/A

How would you rate your current level of function during you usual activities of dailyliving from 0 to 100 with 100 being your level of function prior to your foot or ankleproblem and 0 being the inability to perform any of your usual daily activities.

FAAM - SPORTS SUBSCALE

Please Answer every question with one response that most closely describes your condition within the past week.
If the activity in question is limited by something other than your foot or ankle mark “Not Applicable” (N/A).

Because of your foot and ankle how much difficulty do you have with:

1. Running

- No Difficulty
- Slight Difficulty
- Moderate Difficulty
- Extreme Difficulty
- Unable to do
- N/A

2. Jumping

- No Difficulty
- Slight Difficulty
- Moderate Difficulty
- Extreme Difficulty
- Unable to do
- N/A

3. Landing

- No Difficulty
- Slight Difficulty
- Moderate Difficulty
- Extreme Difficulty
- Unable to do
- N/A

4. Starting and stopping quickly

- No Difficulty
- Slight Difficulty
- Moderate Difficulty
- Extreme Difficulty
- Unable to do
- N/A

5. Cutting/lateral movements

- No Difficulty
- Slight Difficulty
- Moderate Difficulty
- Extreme Difficulty
- Unable to do
- N/A

6. Low impact activities

- No Difficulty
- Slight Difficulty
- Moderate Difficulty
- Extreme Difficulty
- Unable to do
- N/A

7. Ability to perform activity with your normal technique

- No Difficulty
- Slight Difficulty
- Moderate Difficulty
- Extreme Difficulty
- Unable to do
- N/A

8. Ability to participate In your desired sport as long as you would like

- No Difficulty
- Slight Difficulty
- Moderate Difficulty
- Extreme Difficulty
- Unable to do
- N/A

How would you rate your current level of function during your sports related activitiesfrom 0 to 100 with 100 being your level of function prior to your foot or ankle problemand 0 being the inability to perform any of your usual daily activities?

Overall, how would you rate your current level of function?

Marcar apenas uma oval.

- Normal
- Nearly normal
- Abnormal
- Severely Abnormal
