# Supplementary material for: Validation of Foot and Ankle Ability Measure (FAAM) and the Foot and Ankle Outcome Score (FAOS) in individuals with chronic ankle instability: a cross-sectional observational study
Source: J Orthop Surg Res. 2022 Jan 21;17:38. doi: 10.1186/s13018-022-02925-9 (PMC8781557; doi:10.1186/s13018-022-02925-9)
Supplement: Supplementary file 6 — Additional file 6. Foot and Ankle Ability Measure (FAAM) questionnaire - Brazilian version. [file 13018_2022_2925_MOESM6_ESM.pdf]

FOOT AND ANKLE ABILITY MEASURE (FAAM)- SUBESCALA DE ATIVIDADE DE VIDA DIÁRIA - BRAZILIAN VERSION

Por favor, responda todas as questões, indicando uma única alternativa que melhor descreva sua condição na ÚLTIMA SEMANA.
Se a atividade a ser respondida está limitada por algum outro motivo que não esteja relacionado com seu problema no pé ou tornozelo, marque NÃO SE APLICA (N/A)

Por causa do seu problema no tornozelo ou pé, quanta dificuldade você tem em:

1. Ficar em pé

- Nenhuma dificuldade
- Pouca/leve dificuldade
- Moderada dificuldade
- Extrema dificuldade
- Incapaz de fazer
- N/A

2. Caminhar no plano, em superfície regular

- Nenhuma dificuldade
- Pouca/leve dificuldade
- Moderada dificuldade
- Extrema dificuldade
- Incapaz de fazer
- N/A

3. Caminhar no plano, em superfície regular, descalço

- Nenhuma dificuldade
- Pouca/leve dificuldade
- Moderada dificuldade
- Extrema dificuldade
- Incapaz de fazer
- N/A

4. Subir morro

- Nenhuma dificuldade
- Pouca/leve dificuldade
- Moderada dificuldade
- Extrema dificuldade
- Incapaz de fazer
- N/A

5. Descer morro

- Nenhuma dificuldade
- Pouca/leve dificuldade
- Moderada dificuldade
- Extrema dificuldade
- Incapaz de fazer
- N/A

6. Subir escada

- Nenhuma dificuldade
- Pouca/leve dificuldade
- Moderada dificuldade
- Extrema dificuldade
- Incapaz de fazer
- N/A

7. Descer escada

- Nenhuma dificuldade
- Pouca/leve dificuldade
- Moderada dificuldade
- Extrema dificuldade
- Incapaz de fazer
- N/A

8. Caminhar no plano, em superfície irregular

- Nenhuma dificuldade
- Pouca/leve dificuldade
- Moderada dificuldade
- Extrema dificuldade
- Incapaz de fazer
- N/A

9. Subir e descer meio-fio

- Nenhuma dificuldade
- Pouca/leve dificuldade
- Moderada dificuldade
- Extrema dificuldade
- Incapaz de fazer
- N/A

10. Agachar

- Nenhuma dificuldade
- Pouca/leve dificuldade
- Moderada dificuldade
- Extrema dificuldade
- Incapaz de fazer
- N/A

11. Ficar na ponta dos pés

- Nenhuma dificuldade
- Pouca/leve dificuldade
- Moderada dificuldade
- Extrema dificuldade
- Incapaz de fazer
- N/A

12. Começar a caminhar

- Nenhuma dificuldade
- Pouca/leve dificuldade
- Moderada dificuldade
- Extrema dificuldade
- Incapaz de fazer
- N/A

13. caminhar 5 minutos ou menos

- Nenhuma dificuldade
- Pouca/leve dificuldade
- Moderada dificuldade
- Extrema dificuldade
- Incapaz de fazer
- N/A

14. caminhar aproximadamente 10 minutos

- Nenhuma dificuldade
- Pouca/leve dificuldade
- Moderada dificuldade
- Extrema dificuldade
- Incapaz de fazer
- N/A

15. caminhar 15 minutos ou mais

- Nenhuma dificuldade
- Pouca/leve dificuldade
- Moderada dificuldade
- Extrema dificuldade
- Incapaz de fazer
- N/A

16. Atividades domésticas

- Nenhuma dificuldade
- Pouca/leve dificuldade
- Moderada dificuldade
- Extrema dificuldade
- Incapaz de fazer
- N/A

17. Atividades de vida diária

- Nenhuma dificuldade
- Pouca/leve dificuldade
- Moderada dificuldade
- Extrema dificuldade
- Incapaz de fazer
- N/A

18. Cuidado pessoal

- Nenhuma dificuldade
- Pouca/leve dificuldade
- Moderada dificuldade
- Extrema dificuldade
- Incapaz de fazer
- N/A

19. Trabalho leve a moderado que exija caminhar ou ficar em pé

- Nenhuma dificuldade
- Pouca/leve dificuldade
- Moderada dificuldade
- Extrema dificuldade
- Incapaz de fazer
- N/A

20. Trabalho pesado (empurrar/puxar, subir/descer escada, carregar)

- Nenhuma dificuldade
- Pouca/leve dificuldade
- Moderada dificuldade
- Extrema dificuldade
- Incapaz de fazer
- N/A

21. Atividades recreativas

- Nenhuma dificuldade
- Pouca/leve dificuldade
- Moderada dificuldade
- Extrema dificuldade
- Incapaz de fazer
- N/A

De 0 a 100, como você classificaria seu nível atual de função durante atividades de vida diária usuais, sendo 100 correspondente ao mesmo nível de função anterior ao seu problema do seu pé ou tornozelo e 0 (zero) corresponde a incapacidade em desempenhar qualquer das suas atividades diárias usuais?

FAAM -ESCALA DE ESPORTE - BRAZILIAN VERSION

Por favor, responda todas as questões, indicando uma única alternativa que melhor descreva sua condição na ÚLTIMA SEMANA.
Se a atividade a ser respondida está limitada por algum outro motivo que não esteja relacionado com seu problema no pé ou tornozelo, marque NÃO SE APLICA (N/A)

Por causa do seu problema no tornozelo ou pé, quanta dificuldade você tem em:

1. Correr

- Nenhuma dificuldade
- Pouca/leve dificuldade
- Moderada dificuldade
- Extrema dificuldade
- Incapaz de fazer
- N/A

2. Pular

- Nenhuma dificuldade
- Pouca/leve dificuldade
- Moderada dificuldade
- Extrema dificuldade
- Incapaz de fazer
- N/A

3. Amortecer salto

- Nenhuma dificuldade
- Pouca/leve dificuldade
- Moderada dificuldade
- Extrema dificuldade
- Incapaz de fazer
- N/A

4. Arrancar e parar bruscamente

- Nenhuma dificuldade
- Pouca/leve dificuldade
- Moderada dificuldade
- Extrema dificuldade
- Incapaz de fazer
- N/A

5. Realizar passadas laterais rápidas, com mudança brusca de direção

- Nenhuma dificuldade
- Pouca/leve dificuldade
- Moderada dificuldade
- Extrema dificuldade
- Incapaz de fazer
- N/A

6. Atividade de baixo impacto

- Nenhuma dificuldade
- Pouca/leve dificuldade
- Moderada dificuldade
- Extrema dificuldade
- Incapaz de fazer
- N/A

7. Capacidade em desempenhar a atividade com sua técnica normal

- Nenhuma dificuldade
- Pouca/leve dificuldade
- Moderada dificuldade
- Extrema dificuldade
- Incapaz de fazer
- N/A

8. Capacidade em praticar o seu esporte desejado pelo tempo que você gostaria

- Nenhuma dificuldade
- Pouca/leve dificuldade
- Moderada dificuldade
- Extrema dificuldade
- Incapaz de fazer
- N/A

De 0 a 100, como você classificaria seu nível atual de função durante suas atividades esportivas, sendo 100 correspondente ao mesmo nível de função anterior ao seu problema do seu pé ou tornozelo e 0 (zero) corresponde a incapacidade em desempenhar qualquer das suas atividades esportivas usuais?

____

De modo geral, como você classificaria seu nível funcional?

Marcar apenas uma oval.

- ( ) Normal
- ( ) Quase normal
- ( ) Anormal
- ( ) Extremamente anormal
